# The Transition From Undernutrition to Overnutrition Under Adverse Environments and Poverty: The Risk for Chronic Diseases

**DOI:** 10.3389/fnut.2021.676044

**Published:** 2021-04-23

**Authors:** Paola Caroline L. Leocádio, Synara C. Lopes, Ronaldo P. Dias, Jacqueline I. Alvarez-Leite, Richard L. Guerrant, João O. Malva, Reinaldo B. Oriá

**Affiliations:** ^1^Laboratory of Atherosclerosis and Nutritional Biochemistry, Department of Biochemistry and Immunology, Federal University of Minas Gerais, Belo Horizonte, Brazil; ^2^Department of Nutrition, Nursing School, Federal University of Minas Gerais, Belo Horizonte, Brazil; ^3^Laboratory of Tissue Healing, Ontogeny, and Nutrition, Faculty of Medicine, Federal University of Ceará, Fortaleza, Brazil; ^4^Center for Global Health, University of Virginia, Charlottesville, VA, United States; ^5^Coimbra Institute for Clinical and Biomedical Research (iCBR), Faculty of Medicine, University of Coimbra, Coimbra, Portugal; ^6^Center for Innovative Biomedicine and Biotechnology (CIBB), University of Coimbra, Coimbra, Portugal

**Keywords:** malnutrition, obesity, intestinal microbiota, diet, COVID-19, dysbiosis, poverty

Nutritional transition is an important public health issue in developing countries, where switch from undernutrition to overnutrition/obesity is rapidly occurring, often within two or three generations. Such transition is related to changes in lifestyle, with people having more access to western high-caloric diets. In developing countries, settings of poverty, poor sanitation and hygiene are still common, where children are exposed to numerous enteric pathogens, pollutants, and other biohazards. Populations living under such adverse environments and facing the nutritional transition may have increased risks for chronic illnesses in later life, including diabetes, cardiovascular, and neurodegenerative diseases. This opinion paper summarizes novel findings and recent literature addressing the nutrition transition under adverse environments, including the gut microbiota-brain axis dysfunction and their lasting effects with deleterious consequences for later development.

## Dietary Changes in Developing Countries

According to the World Health Organization (WHO), approximately 462 million adults are underweight, 1.9 billion are overweight or obese, and 2 billion are micronutrient deficient (1). In addition, 38 million children below 5 years of age were overweight or obese in 2019 and more than 340 million children and adolescents (5 to 19 years) were overweight or obese in 2016 (2). Obesity associated with micronutrient deficiency has great repercussions in childhood and deserves special attention in middle and low income countries (3), which may be even more aggravated by poverty and unbalanced diets.

In developing countries, lifestyle changes amidst the disarray of urbanization, increasing poverty (with proliferating shantytowns, poor sanitation, and hygiene), crowding, and altered dietary habits. Continuous exposure to harmful pollutants (mercury, lead, arsenic, asbestos etc.) and environmental pathogens may accumulate and lead to a detrimental exposome throughout life with long-term health consequences (4).

In developing countries with emerging economies, a double burden of malnutrition (DBM) often occurs even in impoverished areas. The DBM is defined by WHO as “the coexistence of undernutrition along with overweight, obesity or diet-related non-communicable diseases (NCD), within individuals, households and populations, and across the life-course” and may be aggravated by poor environmental circumstances and genetic predispositions (1). Although a large proportion of deaths among children under five are attributed to malnutrition, overweight and obesity in this age group and older are on the rise (5). The increased risk of NCD related with the nutritional transition in developing countries is a public health concern, due to the potential economic impact and oversaturation of the health system infrastructure (6).

Furthermore, when nutritional deprivation or infection occurs early in life, such as in the prenatal phase and/or up to 3 years of age, the individual undergoes metabolic changes that can lead to greater susceptibility to developing obesity as an adult (7). DBM can cause long-term effects, especially when their components develop early, and each of them can increase the chances of the other occurring (8).

The transition to hypercaloric diets is a global health concern. Hypercaloric intake is characterized by high-carbohydrate and fat consumption, both leading to obesity. Studies have shown that maternal obesity during pregnancy can lead to overweight and other metabolic effects in the offspring through epigenetic mechanisms, such as DNA methylation (9). The coexistence of these opposing nutritional patterns reflects, in part, social and economic inequalities. Other factors such as the increased life expectancy also contribute, as elderly populations are more vulnerable to malnutrition given their psychological, social and health-related risks and chronic diseases (10).

## Impact of Nutritional Transition in Developing Countries

Several studies point to the effects of fetal programming and maternal and environmental factors during early life on the development of diseases in adulthood (11–16). Maternal nutrition even before pregnancy can affect the development of the fetus with later risk for cardiovascular/metabolic diseases (12). Early undernutrition followed by later overweight increases the risk of NCD, imposing a high metabolic load on a reduced or altered capacity for homeostasis. In women, early childhood undernutrition increases the risk of complications in childbirth later in life (13). In countries with poor sanitary conditions, the occurrence of the APOE4, a recognized gene associated with increased risk for acquiring Alzheimer's disease, may favor maternal fertility and promote protection against childhood diarrhea, while possibly increasing the risk of NCD with aging (14). In animal models, maternal obesity affects leptin mRNA expression and its peak in offspring (15). Hyperleptinemia leads to leptin resistance in neonates, favoring hyperphagia and can permanently affect the regulation of appetite (16). These trajectories throughout life are shaped both by driving factors of society–that is, rapid changes in diets, dietary norms and patterns of physical activity–and by broader ecological factors, such as pathogen exposure (8).

In the example of Brazil, the most recent National Health Survey (17) pointed out that 60.3% of the population over 18 years old is overweight, while underweight reaches 1.6% of the inhabitants over 25 years old. A systematic review (18) pointed out that the prevalence of underweight was approximately 10% in children from different regions of the country, reaching 21% due to social disparities. The prevalence of overweight was also approximately 10%, with a reduction to 6% in populations with social inequality.

The increase in the body mass index (BMI) in the population has grown significantly not only in Brazil but in other low- and middle-income countries around the globe, justifying the increase in the incidences of NCD. Yiengprugsawan et al. (19) studied the populations of Brazil and Thailand because both went through similar economic transitions with the hypothesis of the existence of a relationship between BMI and socioeconomic status. In this study, socioeconomic status was directly related to the increase in overweight and obesity in males. In women, an inverse pattern was observed for both cohorts throughout the study period (19).

A more recent survey conducted in Greece after the economic crisis, showed that there are still inequalities in the nutritional level. The DBM is present in populations dependent on the government's food assistance program. The food of the neediest people is unfortunately characterized by a high consumption of processed foods containing refined sugar and a scarce intake of fruits and vegetables. Greece is also undergoing a nutritional transition phase initiated by the economic crisis that occurred 10 years ago and that today still affects the poorest population (20).

## Dietary Imbalance and Inflammation: From the Gut to the Brain

The dynamic gut microbial ecosystem is directly influenced by the diet and by its bidirectional interaction with the health of the host. In the context of DBM, literature points out that changes in the microbiota can be profoundly influenced by both undernutrition and obesity (5). Long-lasting effects of undernutrition in early life can be attributed to interconnected biological pathways, involving imbalance of the gut microbiome, inflammation, metabolic dysregulation, and impaired insulin signaling (8).

The immaturity of the microbiota, the increased burden of enteric pathogens and the dysfunction of the intestinal barrier are associated with systemic inflammation in early life, that may have lasting disabling effects (21). Contaminated environments, early childhood diarrhea and enteric pathogens have been associated with intestinal dysbiosis, increased risk of developing obesity (22) and neurodegenerative diseases (23, 24). Studies have shown the impact of diet and obesity on gut microbiota. High-fat diets may promote dysbiosis (25) and the gut microbiota was stated as a further contributing factor to pathophysiology of obesity by influencing insulin resistance and systemic inflammation (26). Among obese adults with similar BMI, those with greater dysbiosis have a higher risk of NCD (27). Some have suggested that changes in the microbiota promoted by SARS-CoV-2 infection associated with dietary changes may exacerbate present and future health risks (5).

Moreover, the relationship between COVID-19 and nutrition is reciprocal. Both, obesity and micronutrient deficiencies increase the risk and aggravates the evolution of COVID-19, which, in turn, due to its devastating effect, leads to undernutrition. Moreover, social distancing leads to physical inactivity, less frequent sun exposure (reducing stokes of vitamin D), increases stress and reduces medical visits for non-related COVID-19 symptoms (28).

Both undernutrition and obesity may induce low-grade systemic inflammation (29, 30). The adipose tissue secretes several inflammatory mediators and these molecules can exert metabolic, cardiovascular and hepatic effects, among others, favoring the development of NCD (31). In the elderly, the increase in systemic inflammation indicators is related to atherosclerosis, diabetes, neurodegenerative diseases, and carcinogenesis, however it is still not entirely clear why low-grade chronic inflammation is associated with the development of chronic diseases (32).

It has been increasingly recognized that imbalanced diets may be *per se* an proinflammatory factor. The literature presents accumulating evidence that points to the connection between diet, inflammation and NCD (33). These data are essential to find out how obesity is outlined and impacts on the emerging metabolic disorders. The diet lipid content profile can modulate pro-inflammatory genes expression (34). Prolonged consumption of a high-fat diet promotes an increase in inflammatory mediators in the central nervous system and peripheral tissues including liver and adipose tissue (35). Interestingly, (44) observed greater lipid deposition in animals that consumed a diet rich in fat but found a higher concentration of inflammatory mediators in animals that received a diet rich in carbohydrates.

One of the recently studied molecules in non-alcoholic fatty liver disease associated with obesity, involving hyperlipidemia, hyperglycemia and insulin resistance is perilipin-2, a cytoplasmic protein that covers lipid droplets (36). Kern et al. (37) observed greater expression of the protein in obese humans, not associated with an increase in inflammatory mediators and insulin resistance. In animals fed a high-fat diet, the deletion of the perilipin-2 gene prevented obesity, inflammation of adipose tissue, insulin resistance and steatosis (38). Perilipin-2 also appears to play a role in intestinal dysbiosis promoted by a high-fat diet (39).

In summary, dietary changes observed in DBM can contribute to the development of NCD, calling attention for the utmost need to study and implement actions that can mitigate its effects.

## What Can be Done to Reduce the Impact of the Nutritional Transition on the Development of Chronic Diseases?

From observing the effects of the nutritional transition on health and quality of life, especially in countries facing DBM–in which the health system needs to cope with great demands for both addressing poor diet and sanitation in early life as well as the costly management of chronic diseases as adults age (40), interventions are needed to reduce these worrisome impacts.

Interventions, programs, and policies with the potential to simultaneously reduce DBM are called double-duty actions: improving maternal and child nutrition, encouraging breastfeeding, school feeding policies and programs and marketing regulations (41). A scoping review concludes that there are few studies evaluating the effect of these actions to reduce both undernutrition and overnutrition (42). Recent work criticizes double-duty actions, suggesting expansion of the groups contemplated, for example including women of reproductive age in the pre and post-pregnancy periods, expanding the focus of nutrition to general health. Still, some determinants associated with DBM are not considered, such as better access to potable water, which raises the question of the need to revisit and update the current intervention strategies (43).

## Conclusion

The double burden posed by early childhood undernutrition with later life continued poor, high fat diets may have lasting consequences that need to be addressed by sanitary authorities. As the health access improves and life expectancy increases in developing countries, the compound effect of early-life DBM in aging populations even more raises the concern and thus requires more attention by health policies.

In times of COVID-19 pandemic, exacerbation of DBM is foreseen, with likely increased rates of undernutrition (due to micronutrient deficiency, diet imbalance, e.g., vitamin D deficiency) and obesity (food choices, lockdowns, and restrictions on mobility) worldwide, but in low and middle income countries such lifestyle changes, when compounded with adverse environments and poverty may have a profound impact for health and likely increasing the risk for acquiring/aggravating chronic diseases (43). This opinion paper calls for more awareness of this public health issue and the need for long-term cohort studies to follow-up and bring innovative strategies to ameliorate potentially lasting effects of the nutrition transition, especially under adverse environments.

## Author Contributions

All authors have equally contributed to the work and approved it for publication.

## Conflict of Interest

The authors declare that the research was conducted in the absence of any commercial or financial relationships that could be construed as a potential conflict of interest.
